# Natural Compounds from the Marine Brown Alga *Caulocystis cephalornithos* with Potent In Vitro-Activity against the Parasitic Nematode *Haemonchus contortus*

**DOI:** 10.3390/pathogens9070550

**Published:** 2020-07-09

**Authors:** Aya C. Taki, Robert Brkljača, Tao Wang, Anson V. Koehler, Guangxu Ma, Jill Danne, Sarah Ellis, Andreas Hofmann, Bill C. H. Chang, Abdul Jabbar, Sylvia Urban, Robin B. Gasser

**Affiliations:** 1 Department of Biosciences, Melbourne Veterinary School, Faculty of Veterinary and Agricultural Sciences, The University of Melbourne, Parkville, VIC 3010, Australia; aya.taki@unimelb.edu.au (A.C.T.); tao.wang1@unimelb.edu.au (T.W.); anson.koehler@unimelb.edu.au (A.V.K.); guangxum@student.unimelb.edu.au (G.M.); a.hofmann@structuralchemistry.org (A.H.); bchang@yourgene.com.tw (B.C.H.C.); jabbara@unimelb.edu.au (A.J.); 2 School of Science (Applied Chemistry and Environmental Science), RMIT University, Melbourne, VIC 3001, Australia; robert.brkljaca@monash.edu; 3 Peter MacCallum Cancer Centre, Melbourne, VIC 3010, Australia; jill.danne@petermac.org (J.D.); sarah.ellis@petermac.org (S.E.)

**Keywords:** algae, *Caulocystis cephalornithos*, natural compound, anthelmintic, in vitro-activity, *Haemonchus contortus*

## Abstract

Eight secondary metabolites (**1** to **8**) were isolated from a marine sponge, a marine alga and three terrestrial plants collected in Australia and subsequently chemically characterised. Here, these natural product-derived compounds were screened for in vitro-anthelmintic activity against the larvae and adult stages of *Haemonchus contortus* (barber’s pole worm)—a highly pathogenic parasitic nematode of ruminants. Using an optimised, whole-organism screening system, compounds were tested on exsheathed third-stage larvae (xL3s) and fourth-stage larvae (L4s). Anthelmintic activity was initially evaluated on these stages based on the inhibition of motility, development and/or changes in morphology (phenotype). We identified two compounds, 6-undecylsalicylic acid (**3**) and 6-tridecylsalicylic acid (**4**) isolated from the marine brown alga, *Caulocystis cephalornithos,* with inhibitory effects on xL3 and L4 motility and larval development, and the induction of a “skinny-straight” phenotype. Subsequent testing showed that these two compounds had an acute nematocidal effect (within 1–12 h) on adult males and females of *H. contortus*. Ultrastructural analysis of adult worms treated with compound **4** revealed significant damage to subcuticular musculature and associated tissues and cellular organelles including mitochondria. In conclusion, the present study has discovered two algal compounds possessing acute anthelmintic effects and with potential for hit-to-lead progression. Future work should focus on undertaking a structure-activity relationship study and on elucidating the mode(s) of action of optimised compounds.

## 1. Introduction

Parasitic helminths (worms) cause substantial morbidity and mortality in human and animals. The World Health Organization (WHO) estimates that more than 1.5 billion people (24% of the world’s population) are infected with soil-transmitted helminths worldwide [1]. Members of the Phylum Nematoda infect livestock and cause the most prominent losses to food production globally [2]. In particular, the barber’s pole worm, *Haemonchus contortus* (order Strongylida) is a highly pathogenic nematode, primarily of ruminants, and has a global distribution [3]. This blood-feeding worm causes anaemia, associated complications and death in severely affected animals [4] and major productivity and economic losses to farmers and livestock industries [5,6]. Currently, the control of *H. contortus* and related nematodes relies on the use of a limited number of anti-parasitic drugs and their excessive and often uncontrolled use has led to widespread resistance in these worms against these drugs (within as few as two years) [7,8], even to those most-recently introduced into the commercial market (e.g., monepantel) [9,10,11]. The relatively rapid emergence (within 5–10 years) of resistance in gastrointestinal nematode populations [7] means that there is a continued need for the discovery of new compounds with modes/mechanisms of action which are distinct from those presently available on the commercial market [12,13].

Although there has been a major focus on synthetic compounds as anthelmintics [8,14], recent studies have shown that natural compounds from both terrestrial and marine environments have shown considerable promise [15,16,17,18,19] due to the diverse biologically active chemotypes in nature [20]. Current estimates indicate that there are >400,000 land plant species and ≥44,000 species of algae [21,22]. Australia offers one of the most unique and diverse ecosystems, and chemical investigations of native specimens have allowed the isolation and discovery of numerous novel and unique natural compounds from terrestrial or marine environments [23,24]. Some recently discovered natural product scaffolds exhibit antiparasitic, antimicrobial, anti-inflammatory and/or anti-cancer activity [25,26,27,28].

Recent studies by our research team [29,30,31,32] have been focused on isolating secondary metabolites from marine sponges, algae and terrestrial plants in Australia. Some of these natural compounds include the pentaprenylated *p*-quiniol (compound **1**) and furospinosulin-1 (**2**) from *Dactylospongia* sp. (mustard sponge), 6-undecylsalicyclic acid (**3**) and 6-tridecylsalicylic acid (**4**) from *Caulocystis cephalornithos* (marine brown alga), 6-hydroxy-2,5-dimethoxy-9-phenylphenalen-1-one (**5**) from *Haemodorum spicatum* (bloodroot), fuliginosin A (**6**) and anigopreissin A (**7**) from *Macropidia fuliginosa* (black kangaroo paw), and acacetin (**8**) from *Agastache rugosa* (Korean mint). These compounds have been shown to possess anti-inflammatory, anti-cancer and/or antimicrobial properties [29,33,34,35,36,37]; the latter properties suggest that some of these compounds might also have activities against pathogens such as parasites. In the present study, we explored the activity of select secondary metabolites against *H. contortus* using established in vitro methods, with the aim of identifying candidate compounds to optimise and develop as anthelmintic drugs. 

## 2. Results

### 2.1. Reduction in Larval Motility and/or Development, and Phenotypic Alteration

**Figure 1:**
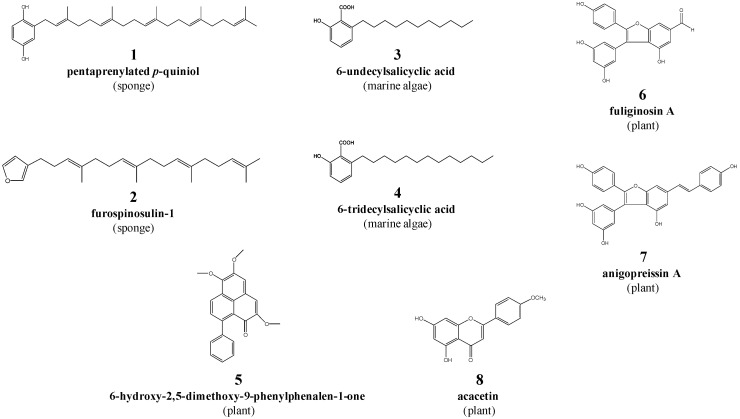
The structures of eight natural compounds (**1** to **8**) investigated in this study.

**Figure 2:**
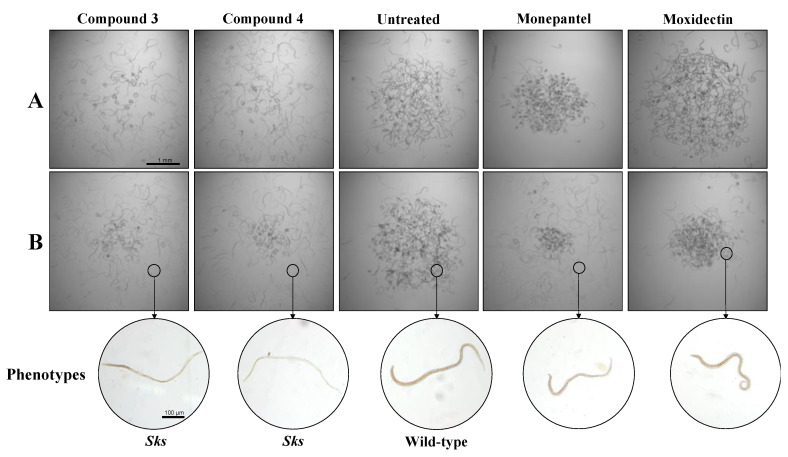
Testing of compounds **3** and **4** on the fourth-stage (L4s) of *Haemonchus contortus*. Representative images (25-times magnification) of xL3s cultured to L4s over seven days in the presence of individual test compounds (**3** and **4**) and control compounds (moxidectin and monepantel) at 20 μM in primary screen (panel **A**), and of L4s exposed over 72 h to the same individual test compounds (1.56 µM each) control compounds (moxidectin and monepantel) at 50 μM in a dose-response assay (panel **B**). Compounds **3** and **4** induced a skinny-straight (*Sks*) phenotype in L4s in comparison to the wild-type phenotype control (cultured in LB* + 0.5\% dimethyl sulfoxide). Worms representative of individual (morphological) phenotypes are encircled (bottom).

Initial screening of the eight compounds (each at 20 µM; Figure 1) on *H. contortus* suggested that compounds **7** and **8** each reduced xL3 motility by ~50% after 72 h, but this effect was not reproducible, and showed that compounds **3** and **4** each induced an abnormal phenotype in >90% of L4s exposed for seven days (Figure 2A). The latter phenotype (designated as “skinny-straight”, *Sks*) was characterised by thin, straight larvae with reference to larvae exposed to DMSO alone (which had no apparent abnormality; cf. [38]).

**Figure 3:**
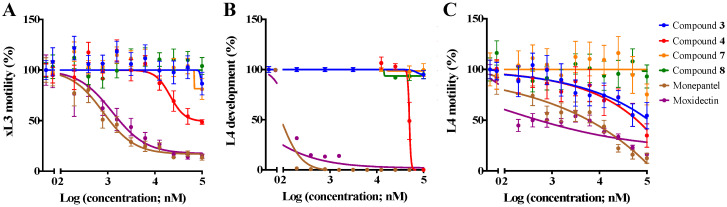
In vitro-activity of compounds against exsheathed third-stage larvae (xL3) and fourth-stage larvae (L4) of *Haemonchus contortus*. Dose-response curves of the individual test compounds (**3**, **4**, **7** and **8)** and control compounds (monepantel and moxidectin) assessing the inhibition of xL3-motility at 72 h (panel **A**), xL3s cultured to L4s over seven days (panel **B**) and L4-motility at 72 h (panel **C**). Data points represent three independent experiments conducted in triplicates; the mean ± standard error of the mean (SEM).

A subsequent assessment of compounds **3**, **4**, **7** and **8** in dose-response assays revealed that compound **4** was the most potent at reducing xL3-motility by 51.3 ± 0.4% at 100 μM after 72 h (IC_50_ = 20.3 ± 0.9 μM; Figure 3A). Compound **4** (at 100 μM) also inhibited the development of xL3s to the L4 stage over seven days (IC_50_ = 40.7 ± 7.4 μM; Figure 3B). Compounds **3** and **4** (each at 100 μM) each inhibited L4-motility by 46% and 65%, respectively, within 72 h (Figure 3C), and induced an *Sks*-phenotype in >90% of L4s (72 h) at concentrations as low as 0.78–1.56 μM (Figure 2B), similar to that observed in L4s exposed to monepantel at 50 µM. 

### 2.2. Acute Nematocidal Activity on Adults

**Figure 4:**
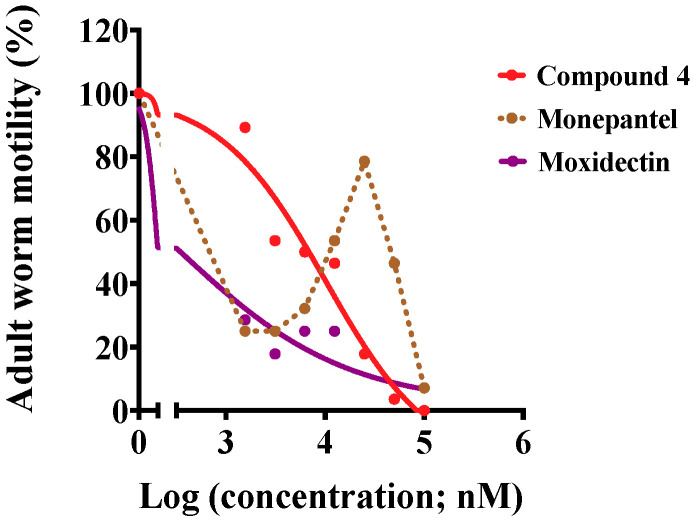
In vitro-activity of compound **4** against adult females of *Haemonchus contortus* at 1 h. Dose-response curves of the effects of compound **4** and each of the positive controls (monepantel and moxidectin) on the inhibition of adult female motility at 1 h. Data points and solid trend-lines fitted using a variable slope four-parameter equation for compound **4** and moxidectin; data points connected with a dotted line for monepantel.

Compounds **3** and **4** were tested separately on adult females and males of *H. contortus*. Already after 6 h of exposure to 50 μM and 100 μM of each of the two compounds, 90% and 100% of females, respectively, were immotile compared with slight motility in positive-control wells (monepantel or moxidectin, at the same concentrations) (Table 1). Under the same conditions, 100% of males were immotile for both concentrations, compared with only slight motility in positive-control wells (Table 1). Compound **4** was assessed further in a dose-response assay, and inhibited the motility of adult females with the IC_50_ value of 10.1 μM after 1 h of exposure (Figure 4), which remained consistent for a further 3 h-period. In the same assay, moxidectin affected adult females similarly over time with an IC_50_ value of 0.3 μM, while monepantel was most potent at two concentration ranges (1.56–6.25 μM and 50–100 μM; Figure 4).

**Figure 5:**
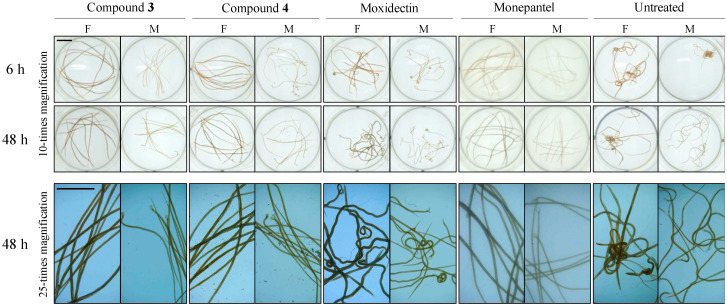
Adult *Haemonchus contortus* exposed to individual test compounds (**3** and **4**) and control compounds (monepantel and moxidectin) for 6 h or 48 h. Wells containing ten adult female (F) and male (M) were exposed to 100 μM of each of test and control compound, and imaged to examine worm morphology with reference to untreated control (1% dimethyl sulfoxide) and two positive controls; monepantel and moxidectin. For both sexes, all worms exposed to each test compound exhibited a “straight” phenotype at 6 h and 48 h. Worms (irrespective of sex) exposed to monepantel had a similar phenotype, but those exposed to moxidectin exhibited a distinct phenotype with a coiled posterior end (in males) and partly-coiled/twisted mid-section (in females) at 6 h and a “wavy”-phenotype by 48 h. Scale bar = 5 mm.

An assessment of worm morphology revealed that adult *H. contortus* of each sex exposed to each compound (**3** and **4)** exhibited a “straight” phenotype at 6 h (Figure 5), similar to that seen in worms exposed to monepantel alone (same concentration), with no morphological alteration seen in worms exposed for 48 h (Figure 5). A distinctly different phenotype (coiled/turned mid-section in female, and coiled posterior end in male) was exhibited by worms exposed to moxidectin for 6 h, which ‘transformed’ into a “wavy” phenotype by 48 h (see Figure 5). 

**Figure 6:**
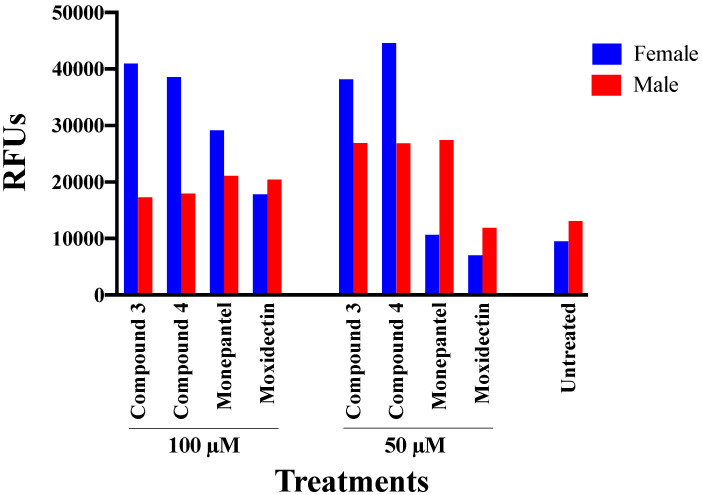
Viability of adult female and male worms of *Haemonchus contortus*. The viability of adult worms (n = 10 per well) exposed to each test compound (50 µM or 100 µM) was compared with that of worms (n = 10 per well) exposed to monepantel, moxidectin (control compounds) at the same concentrations or 1% dimethyl sulfoxide alone (no compound), for 12 h. Following SYTOX™ Green nucleic acid staining, the (normalised) viability of worms was measured (relative fluorescence units, RFUs).

The viability of adult worms exposed to each compound **3** and **4** for 12 h was evaluated in the fluorescence-based assay. Female exposed to 50 μM of each compound **3** and **4** were markedly less viable (38,202–44,581 RFUs) than of those exposed to each of the positive-control compounds (7014–10,665 RFUs; similar to untreated control) (Figure 6), with less variation in viability at 100 μM than 50 µM. Adult males exposed to 50 and 100 μM of each of these two compounds were markedly less viable than untreated control worms and similar to worms exposed to the same concentrations of monepantel.

### 2.3. Subcuticular Tissue Damage in Adult Worms

**Figure 7:**
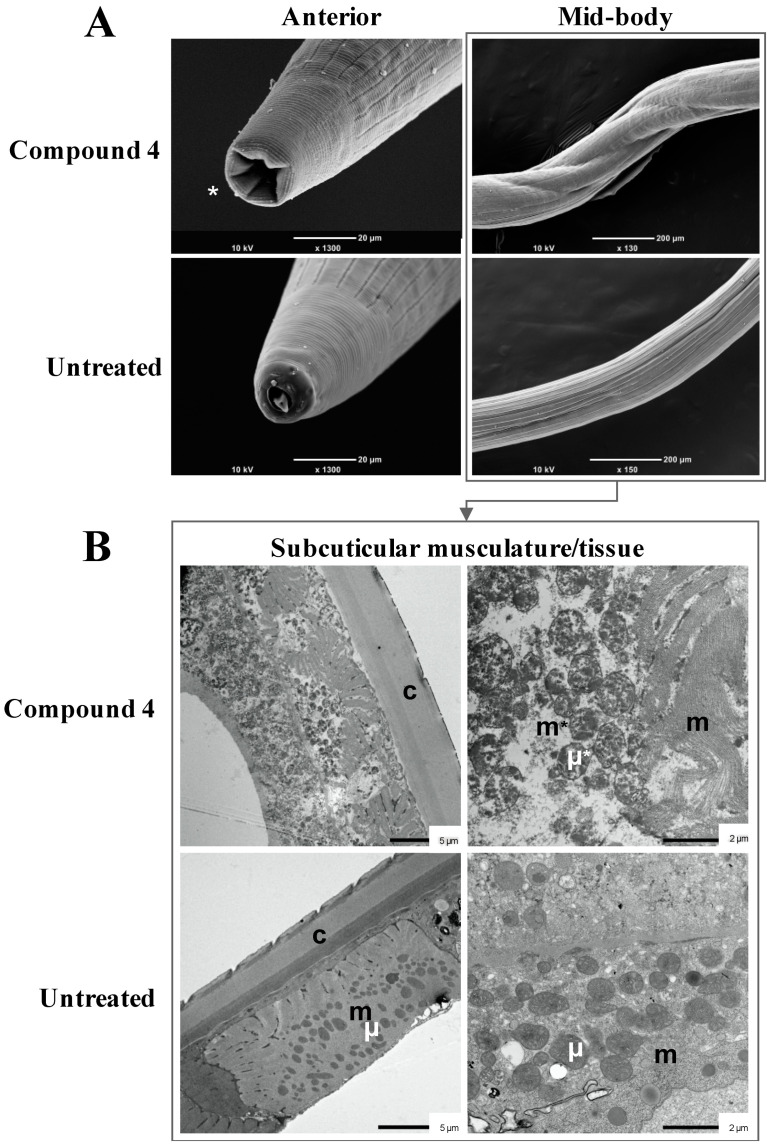
Scanning and transmission electron microscopy (SEM and TEM) of adult specimens of *Haemonchus contortus* exposed to 100 µM of compound **4** or 1% dimethyl sulfoxide alone (no compound) for 48 h in vitro. SEM micrographs (panel **A**) reveal a distinct shrinkage of worms exposed to compound **4** and an associated retraction of the mouth (*) and pronounced the barber’s pole appearance in the mid-body section. TEM micrographs (panel **B**) show shortened and compressed annuli (ridges) on cuticle (c) and a distinct vacuolisation in some musculature (m*) and mitochondria (µ*) in exposed worms, evident as electro-lucent (e) areas, and no observed alterations in the musculature (m), mitochondria (µ) or cuticle (c) of no-compound control worms.

The “straight” phenotype induced in adult *H. contortus* by compound **4** was examined by SEM and TEM (Figure 7). This phenotype was characterised by a retraction of the mouth compared with the wild-type phenotype, in which the buccal capsule and dorsal lancet [40,41] were readily visible (Figure 7A). The barber’s pole appearance of affected worms was pronounced due to shrinkage, particularly in the mid-body section (Figure 7A) and was exacerbated in worms exposed to compound **4** for 48 h compared with 6 h (not shown). The cuticle appeared as intact after compound treatment, but annuli (ridges) on the cuticle appeared as shortened and compressed in ultra-thin transverse mid-body sections (see Figure 7B). Subcuticular tissues (hypodermis) and body musculature were severely damaged/disintegrated and associated with a release and alteration of mitochondria (Figure 7B). Cytoplasmic vacuolisation and disruption of mitochondrial membranes were evidenced by electro-lucent areas within tissues (Figure 7B). None of these pathological changes was evident in untreated worms. The cellular damage seen in female worms and male worms exposed to compound **4** was the same, and similar to that seen in worms treated with compound **3** (not presented).

## 3. Discussion

Compounds **3** (6-undecylsalicylic acid) and **4** (6-tridecylsalicylic acid) from *C. cephalornithos* (marine brown alga) had appreciable in vitro-activities against the larval and adult stages of *H. contortus*. Compounds **3** and **4** (100 μM) each inhibited L4-motility by 46% and 65%, respectively, within 72 h (Figure 3C), and induced a *Sks*-phenotype in L4s (72 h) at concentrations as low as 0.8 μM. The potency of compound **4** to inhibit the motility of L4 was greater than its effect on xL3, which might be explained by the well-developed mouth and pharynx in the L4 stage, resulting in more rapid oral uptake into and accumulation of the compound in the worm (cf. [42,43]). The *Sks*-phenotype in L4s and the substantial subcuticular tissue destruction in the adult stage of *H. contortus* bode well for further work on this and similar chemical scaffolds. 

The major tissue damage and associated cytoplasmic and mitochondrial vacuolisation seen in the adult stage exposed to compound **4** and the rapid decline in the worms’ motility and viability indicate a marked disruption of cellular physiology and functions, possibly resulting from a direct or indirect effect on mitochondria, an imbalance of fluid exchanges between the pseudo-coelomic space and muscle and/or an abnormal osmotic pressure within the worm. Surprisingly, these tissue and cellular alterations are similar to those seen in adults of *H. contortus* collected from goats fed tannin-rich plants [44]. The extracts of these plants, however, caused the formation of lesions on the cuticle, including mouth, of *H. contortus* in vivo and in vitro [45,46], presenting a distinctly different phenotype from that induced by compound **4**. Given that these compounds are very distinct in structure, it is possible that the cellular damage seen in the worm is the result of a stress-response due to the exposure to the compound, rather than the result of a highly specific mode of action. It would be interesting to explore the molecular or mechanistic determinant(s) of the subcuticular tissue damage and apparently associated cellular destruction and vacuolisation, and to test the hypothesis that these alterations in the worm are linked to the malfunction of mitochondria caused by a reduced expression of one or more genes (e.g., cytochrome *c* oxidase, cytochrome *b*, ATP synthase and NADH dehydrogenase) and subsequent apoptosis [47]. The use of transcriptomics and/or proteomics (cf. [17,48,49,50]) might assist in testing this hypothesis and in elucidating the patho-molecular, -biochemical and/or -physiological mechanism(s).

The higher potency of compound **4** than **3** on both larval and adult stages of *H. contortus* might be explained, at least partially, by its extended alkyl chain (cf. Figure 1). The longer chain leads to higher lipophilicity [51], thereby possibly allowing for a more efficient uptake into the worm. Compound **4** (6-tridecylsalicylic acid from *C. cephalornithos*) was first isolated and described for its anti-inflammatory property in 1980 [52] using a murine model [33]. Since then, structurally-related analogues of 6-tridecylsalicylic acid have been discovered in *C. cephalornithos* (see [34,52]); however, their activities have not yet been investigated. Although 6-undecylsalicylic acid and 6-tridecylsalicylic acid were originally isolated from a southern Australian specimen of *C. cephalornithos*, these compounds were identified recently in another species of marine brown alga, *Sargassum decipiens* [30], but not assessed for nematocidal or nematostatic activity. The potent effect of compound **4** on *H. contortus* warrants future work to evaluate the antiparasitic activities/potencies of 6-tridecylsalicylic acid and its analogues from a range of species of marine algae. 

Compound **4** from *C. cephalornithos* and a series of analogues should be tested for their absorption, distribution, metabolism, excretion and toxicity (ADMET) profiles [53]. Initially, we propose that in vitro-cytotoxicity testing be carried out using HepG2 human hepatoma cells [54], as there is presently no information for 6-tridecylsalicylic acids. Interestingly, a structurally-similar molecule, gingkolic acid from *Ginkgo biloba* L., has selective toxicity on tumorigenic cells, including HepG2 and human laryngeal carcinoma (HEp-2) cells, but no toxic effect on non-tumour cells [55,56]. Ginkgolic acid has been studied relatively extensively for its therapeutic effects on cryptosporidiosis [57,58], human immunodeficiency virus (HIV) and HIV infection [59], hypertension [60,61] and cancer [56,62]. Furthermore, *G. biloba* L. has long been used in traditional Chinese medicine for treating lung and skin diseases [63,64], and its extracts have been assessed also as a remedy for cognitive impairment treatment in people [65,66]. Based on the licenced use of *G. biloba* extracts [65] and the selective toxicity of ginkgolic acid, it is possible that compound **4** and related compounds from algae, such as *S. decipiens*, might exert toxic effects selectively towards parasitic cells over mammalian host cells. This aspect also warrants investigation.

## 4. Materials and Methods 

### 4.1. Compounds and Preparation 

The eight natural compounds selected for this study are listed in Table 2, and their structures depicted in Figure 1. Samples were extracted using 3:1 methanol (MeOH)/dichloromethane (DCM), followed by sequential partitioning into DCM and MeOH, respectively. The DCM fraction was subjected to flash silica chromatography or reversed-phased semi-preparative high-performance liquid chromatography (HPLC) for compound isolation. The collection, isolation and structural characterisation details of each compound are described in Supplementary File 1.

### 4.2. Procurement of Larval and Adult Stages of H. contortus 

*H. contortus* (Haecon-5 strain) was maintained in experimental sheep [67] in accordance with institutional animal ethics approval (permit no. 1714374; The University of Melbourne). Helminth-free Merino sheep (male; eight months of age) were orally inoculated with 7000 third-stage larvae (L3s) of *H. contortus*. Faecal samples containing *H. contortus* eggs were collected every day from 21 days following inoculation. These samples were incubated at 27 °C for one week to produce L3s [38], which were harvested and sieved through two layers of nylon mesh (20 μm pore size; Rowe Scientific, Doveton, VIC, Australia) to remove debris or dead larvae, and then stored at 10 °C for up to 6 months until use. To produce exsheathed third-stage larvae (xL3s), L3s were incubated in 0.15% (*v*/*v*) sodium hypochlorite for 20 min at 37 °C [38]. Adult *H. contortus* were collected from the abomasa of sheep infected for 10 weeks, washed extensively in phosphate-buffered saline (PBS, pH 7.4) and then in RPMI 1640 media supplemented with 2 mM L-glutamine, 100 U/mL penicillin, 100 μg/mL streptomycin and 0.25 μg/mL amphotericin B (RPMI*; Thermo Fisher Scientific, Scoresby, VIC, Australia), and female and male worms separated immediately prior to the testing of compounds. 

### 4.3. Screening of Compounds on xL3s of H. contortus

Compounds were screened at a concentration of 20 μM on xL3s of *H. contortus*, essentially as described previously [38]. In brief, compounds were dissolved to a stock concentration of 20 mM in dimethyl sulfoxide (DMSO; cat no. 2225; Thermo Fisher Scientific, Scoresby, VIC, Australia), then individually diluted to a final concentration of 20 μM using Luria Bertani broth (LB) supplemented with 100 U/mL of penicillin, 100 μg/mL of streptomycin and 0.25 μg/mL of amphotericin (LB*). LB* + 0.5% DMSO, serving as a negative control, and two distinct positive control compounds [20 μM of each monepantel (Zolvix, Elanco, Greenfield, IN, USA) and moxidectin (Cydectin, Virbac, Carros, France)] were applied (in triplicate) to wells of a 96-well microtiter plates (Corning, Corning, NY, USA), and xL3s (~300/well) then added. Monepantel paralyses *H. contortus* by binding to the ligands of nicotinic acetylcholine receptor subunits expressed in body wall muscle cells [68,69], and moxidectin causes flaccid paralysis by binding to gamma-aminobutyric acid (GABA)-gated ion channels in neurons [70].

Following the incubation of the 96-well microtitre plates containing xL3s for 72 h at 38 °C, 10% (*v*/*v*) CO_2_ and >90% humidity, a video recording (5 s) was taken of each well using a grayscale camera (Rolera bolt sCMOS camera, QImaging Scientific, Tucson, AZ, USA) and a motorised X-Y axis stage (BioPoint 2, Ludl Electronics Products, Hawthorne, NY, USA). Individual videos were processed for a motility index (Mi) using the unique algorithm written in a custom macro and analysed through the program ImageJ (v.2.0.0, Fiji) [38]. Primary screening was performed in triplicate, twice, on separate days. A compound was recorded as having activity if it reduced xL3 motility by ≥70% after 72 h of incubation. Data (Mi) from each assay were normalised to a percentage compared with the positive (moxidectin and monepantel) and negative control (LB* + 0.5% DMSO). 

### 4.4. Dose-Response Assessments of Active Compounds on xL3s and L4s of H. contortus 

Compounds that reduced the motility of xL3 in primary screen (compounds **3**, **4**, **7** and **8**) were verified by determining the half maximum inhibitory concentrations (IC_50_) calculated by the generation of dose-response curves at 72 h. Compounds were serially diluted 2-fold in 50 μL of LB*, starting at a concentration of 100 μM down to 0.76 nM, in 96-well microtitre plates (Corning, Corning, NY, USA). Either xL3s or L4s were added to individual wells containing titrated compounds in 50 μL of LB* at a density of 300 per well. All plates were incubated at 38 °C and 10% (*v*/*v*) CO_2_ with >90% humidity. After 72 h of incubation, the motility was measured and analysed to generate an 18-point dose-response curve. Plates containing xL3s were further incubated for four days and these compounds were also tested for their ability to inhibit the development of xL3s to L4s. The rate of L4 development was evaluated by microscopically assessing the presence or absence of mouth and pharynx [41], and directly compared with negative controls (LB* + 0.5\% DMSO). Monepantel and moxidectin were prepared in the same dilution series and referenced as positive controls. All assays (xL3 motility, L4 motility and L4 development) were performed in triplicate, three times, on separate days. Data (Mi) from each assay were converted to a percentage compared with the negative control (LB* + 0.5\% DMSO). To establish IC_50_ values, compound concentrations were log_10_-transformed, fitted using a variable slope four-parameter equation with constraining the top value to 100% using a least squares (ordinary) fit model using the GraphPad Prism (v. 8.1.0) software (GraphPad Software, San Diego, CA, USA).

### 4.5. Assessment of the Activity of Selected Compounds on Adult H. contortus

The in vitro-activity of two compounds (**3** and **4)** was evaluated using adults of *H. contortus*. Compounds **3** or **4** were added to individual wells of 12-well plates (Corning, Corning, NY, USA) at final concentrations of 50 and 100 μM in phenol-red free RPMI*. Corresponding concentrations of monepantel and moxidectin were included as positive controls, and medium containing 1% DMSO served as an untreated (negative) control. Ten adults of each sex were added to wells and exposed to each compound and each control at 40 °C and 10% (*v*/*v*) CO_2_ with >90% humidity; 6 and 48 h after the exposure, a short video recording (20 s) of each well in each plate was taken and the effect of each compound was assessed based on a reduction of motility. Motility was scored as 3 (“good”), 2 (“low”), 1 (“very low”) and 0 (“no movement”), according to a previous publication [39]. Additionally, compound **4** was tested on females in a seven-point dose-response assay (from 100 μM to 1.56 μM at 2-fold dilutions). Motility was evaluated at 1, 2 and 3 h using the same scoring system. The sum of motility scores for each compound was calculated for each concentration and normalised against the no-compound control (RPMI*+1% DMSO) and calculated as a percentage. To establish IC_50_ values, compound concentrations were log_10_-transformed, fitted using a variable slope four-parameter equation using a least squares (ordinary) fit model using the GraphPad Prism (v. 8.1.0) software. 

At 12 h, the viability of treated and untreated adult worms was evaluated using 1 μM SYTOX™ green nucleic acid stain (Thermo Fisher Scientific, Scoresby, VIC, Australia), by labelling in the dark at 40 °C, 10% (*v*/*v*) CO_2_ and >90% humidity, 4 h prior to examination. For each treatment, ten female or ten male worms of *H. contortus* were dispensed into individual wells of a 48-well plate (Corning, Corning, NY, USA), and the relative fluorescent units (RFUs) in each well measured at 503 nm/ex and 528 nm/em using Biotek Synergy H1 plate reader (Bio Tek Instruments, Winooski, VT, USA). 

### 4.6. Scanning Electron Microscopy (SEM)

Individual adults were examined by SEM. Ten adults per treatment, for each sex, in 1 mL of RPMI* in a 12-well plate, were exposed to 100 μM of compounds **3** or **4** for 6 h and 48 h at 40 °C, 10% (*v*/*v*) CO_2_ and >90% humidity. Adults treated with monepantel, moxidectin or 1% DMSO were also included as controls. Worms were washed with 0.1 M sodium cacodylate trihydrate once and fixed in 2.5% glutaraldehyde and 2% paraformaldehyde for 3 h at room temperature then fixed overnight. On the following day, fixed worms were rinsed three times in 0.1 M sodium cacodylate trihydrate for 10 min, and then post-fixed in 2% osmium tetroxide at room temperature for 1 h. Fixed worms were then rinsed three times in distilled water for 15 min. Following fixation, worms were dehydrated in a graded ethanol series (30%, 50%, 70%, 90% and 100%) for 1 h, dried in a critical point dryer (EM CPD300, Leica, Wetzlar, Germany) overnight, mounted on to aluminium stubs (50 mm) with double-sided sticky tape and then sputter-coated with gold. Adult worms were imaged using a JEOL JCM-6000 Plus NeoScope scanning electron microscope (JEOL, Tokyo, Japan); all worms were examined for each treatment group.

### 4.7. Transmission Electron Microscopy (TEM)

Sections of adult worms were examined for the anatomical alterations induced by compounds using TEM. Individual adults were exposed to 100 μM of compounds **3** or **4** for 48 h, washed and fixed for SEM preparation. Adult worms treated with monepantel, moxidectin or 1% DMSO were included as controls. After fixation, worms were post-fixed in 1% osmium tetroxide and 1.5% potassium ferrocyanide in the dark for 2 h at 22 °C before being rinsed again three times in 0.1 M sodium cacodylate trihydrate for 1 h. The fixed adult worms were washed three times in distilled water for 1 h and dehydrated in a graded ethanol series (50%, 70%, 90%, 95% and 100%) for 2 h on a platform rocker, rinsed two times in 100% acetone for 45 min, rocking, and then embedded in Spurr’s resin. Ultrathin sections were cut with an EM UC7 ultramicrotome (Leica Microsystems GmbH, Wetzlar, Germany) and contrasted with lead citrate and aqueous uranyl acetate. Sections were imaged using a transmission electron microscope (JEOL JEM-1400 Flash, Tokyo, Japan) with an integrated JEOL sCMOS camera (JEOL, Tokyo, Japan).

## 5. Conclusions

We have discovered two compounds (**4** and **3**) from the marine brown alga with relatively potent and acute anthelmintic effects on multiple developmental stages of *H. contortus*. This appears to be the first report of algal compounds with profound effects on both larval and adult stages of an economically-important parasitic nematode. We believe that the 6-tridecylsalicylic acid scaffold identified has potential to be developed as an anthelmintic, provided that increased and selective potency for *H. contortus* can be attained. Future work should focus on undertaking a structure-activity relationship study, on elucidating the mode(s) of action of optimised compounds using molecular tools and defining their ADMET profile(s).

## Figures and Tables

**Table 1:** In vitro-activity of compounds **3** and **4** against adult *Haemonchus contortus*. The effect of individual test compounds (**3** and **4**) on the inhibition of motility was assessed at 6 h against control compounds (monepantel and moxidectin) or 1% dimethyl sulfoxide alone (no compound). Motility of individual worms (n = 10 per treatment for each sex) in individual wells was recorded (20 s) using a video camera and then scored (see footnote). The percentage (%) of worms with a particular motility score is indicated.

Worm Sex	Treatment								
	Compound 3		Compound 4		Monepantel		Moxidectin		Untreated
	50 μM	100 μM	50 μM	100 μM	50 μM	100 μM	50 μM	100 μM	(1% DMSO)
**Female**	1; 10% 0; 90%	0; 100%	1; 10% 0; 90%	0; 100%	3; 80% 2; 20%	2; 20% 0; 80%	2; 30% 1; 30% 0; 60%	1; 10% 0; 90%	3; 90% 2; 10%
**Male**	0; 100%	0; 100%	0; 100%	0; 100%	2; 20% 0; 80%	1; 10% 0: 90%	2; 20% 1; 10% 0; 70%	0; 100%	3; 100%

Scoring system used (cf. [39]): 3: good motility, 2: low motility, 1: very low motility, and 0: no motility

**Table 2:** Information on the eight natural compounds studied.

Compound Code	Compound	Molecular Weight	Origin (Common Name)
1	pentaprenylated *p*-quiniol	450.4	*Dactylospongia* sp. (Mustard sponge)
2	furospinosulin-1	354.3	*Dactylospongia* sp. (Mustard sponge)
3	6-undecylsalicyclic acid	292.2	*Caulocystis cephalornithos* (Marine brown alga)
4	6-tridecylsalicylic acid	320.2	*Caulocystis cephalornithos* (Marine brown alga)
5	6-hydroxy-2,5-dimethoxy-9-phenylphenalen-1-one	346.1	*Haemodorum spicatum* (Bloodroot; plant)
6	fuliginosin A	362.1	*Macropidia fuliginosa* (Black kangaroo paw; plant)
7	anigopreissin A	452.1	*Macropidia fuliginosa* (Black kangaroo paw; plant)
8	acacetin	284.1	*Agastache rugosa* (Korean mint; plant)

## Supplementary Materials

The following are available online at https://www.mdpi.com/2076-0817/9/7/550/s1. Supplementary File 1 describing the collection, extraction and purification of compounds; references [36,37] are cited in this supplementary file.

## References

[1] World Health Organization (WHO) Soil-Transmitted Helminth Infections. www.who.int/news-room/fact-sheets/detail/soil-transmitted-helminth-infections..

[2] Mavrot F., Hertzberg H., Torgerson P. (2015). Effect of gastro-intestinal nematode infection on sheep performance: A systematic review and meta-analysis. Parasit. Vectors.

[3] Gasser R.B., von Samson-Himmelstjerna G. (2016). Haemonchus contortus and Haemonchosis—Past, Present and Future Trends. Adv. Parasit.

[4] Besier R.B., Kahn L.P., Sargison N.D., Van Wyk J.A. (2016). Diagnosis, treatment and management of *Haemonchus contortus* in small ruminants. Adv. Parasitol..

[5] Roeber F., Jex A.R., Gasser R.B. (2013). Impact of gastrointestinal parasitic nematodes of sheep, and the role of advanced molecular tools for exploring epidemiology and drug resistance—an Australian perspective. Parasit. Vectors.

[6] Hotez P.J., Alvarado M., Basáñez M.-G., Bolliger I., Bourne R., Boussinesq M., Brooker S.J., Brown A.S., Buckle G., Budke C.M. (2014). The global burden of disease study 2010: Interpretation and implications for the neglected tropical diseases. PLoS Negl. Trop. Dis..

[7] Kotze A.C., Prichard R.K. (2016). Anthelmintic resistance in *Haemonchus contortus*: History, mechanisms and diagnosis. Adv. Parasitol..

[8] Jiao Y., Preston S., Hofmann A., Taki A., Baell J., Chang B.C.H., Jabbar A., Gasser R.B. (2020). A perspective on the discovery of selected compounds with anthelmintic activity against the barber’s pole worm—where to from here?. Adv. Parasitol..

[9] Mederos A.E., Ramos Z., Banchero G.E. (2014). First report of monepantel *Haemonchus contortus* resistance on sheep farms in Uruguay. Parasit. Vectors.

[10] Van den Brom R., Moll L., Kappert C., Vellema P. (2015). *Haemonchus contortus* resistance to monepantel in sheep. Vet. Parasitol..

[11] Sales N., Love S. (2016). Resistance of *Haemonchus* sp. to monepantel and reduced efficacy of a derquantel/abamectin combination confirmed in sheep in NSW, Australia. Vet. Parasitol..

[12] Geary T.G., Conder G.A., Bishop B. (2004). The changing landscape of antiparasitic drug discovery for veterinary medicine. Trends Parasitol..

[13] Zajíčková M., Nguyen L.T., Skálová L., Stuchlíková L.R., Matoušková P. (2019). Anthelmintics in the future: Current trends in the discovery and development of new drugs against gastrointestinal nematodes. Drug Discov. Today.

[14] Taman A., Azab M. (2014). Present-day anthelmintics and perspectives on future new targets. Parasitol. Res..

[15] Kumarasingha R., Preston S., Yeo T.-C., Lim D.S.L., Tu C.-L., Palombo E.A., Shaw J.M., Gasser R.B., Boag P.R. (2016). Anthelmintic activity of selected ethno-medicinal plant extracts on parasitic stages of *Haemonchus contortus*. Parasit. Vectors.

[16] Mengistu G., Hoste H., Karonen M., Salminen J.-P., Hendriks W.H., Pellikaan W.F. (2017). The in vitro anthelmintic properties of browse plant species against *Haemonchus contortus* is determined by the polyphenol content and composition. Vet. Parasitol..

[17] Preston S., Korhonen P.K., Mouchiroud L., Cornaglia M., McGee S.L., Young N.D., Davis R.A., Crawford S., Nowell C., Ansell B.R.E. (2017). Deguelin exerts potent nematocidal activity via the mitochondrial respiratory chain. FASEB J..

[18] Herath H.M.P.D., Preston S., Jabbar A., Garcia-Bustos J., Taki A.C., Addison R.S., Hayes S., Beattie K.D., McGee S.L., Martin S.D. (2019). Identification of Fromiamycalin and Halaminol A from Australian Marine Sponge Extracts with Anthelmintic Activity against *Haemonchus contortus*. Mar. Drugs.

[19] Mravčáková D., Váradyová Z., Kopčáková A., Čobanová K., Grešáková Ľ., Kišidayová S., Babják M., Dolinská M.U., Dvorožňáková E., Königová A. (2019). Natural chemotherapeutic alternatives for controlling of haemonchosis in sheep. BMC Vet. Res..

[20] Chassagne F., Cabanac G., Hubert G., David B., Marti G. (2019). The landscape of natural product diversity and their pharmacological relevance from a focus on the Dictionary of Natural Products^®^. Phytochem. Rev..

[21] Christenhusz M.J.M., Byng J.W. (2016). The number of known plants species in the world and its annual increase. Phytotaxa.

[22] Lughadha E.N., Govaerts R., Belyaeva I., Black N., Lindon H., Allkin R., Magill R.E., Nicolson N. (2016). Counting counts: Revised estimates of numbers of accepted species of flowering plants, seed plants, vascular plants and land plants with a review of other recent estimates. Phytotaxa.

[23] Simpson B.S., Bulone V., Semple S.J., Booker G.W., McKinnon R.A., Weinstein P. (2016). Arid awakening: New opportunities for Australian plant natural product research. Rangeland J..

[24] Carroll A.R., Copp B.R., Davis R.A., Keyzers R.A., Prinsep M.R. (2019). Marine natural products. Nat. Prod. Rep..

[25] Biva I.J., Ndi C.P., Griesser H.J., Semple S.J. (2016). Antibacterial constituents of *Eremophila alternifolia*: An Australian aboriginal traditional medicinal plant. J. Ethnopharmacol..

[26] Raju R., Gunawardena D., Ahktar M., Low M., Reddell P., Münch G. (2016). Anti-inflammatory chemical profiling of the Australian rainforest tree *Alphitonia petriei* (Rhamnaceae). Molecules.

[27] Gordon S., Wibowo M., Wang Q., Holst J., Davis R.A. (2018). Dihydro-*β*-agarofurans from the Australian rainforest plant *Denhamia celastroides* that inhibit leucine transport in prostate cancer cells. Magn. Reson. Chem..

[28] Kumar R., Duffy S., Avery V.M., Carroll A.R., Davis R.A. (2018). Microthecaline A, a quinoline serrulatane alkaloid from the roots of the Australian desert plant *Eremophila microtheca*. J. Nat. Prod..

[29] Dias D.A., Urban S. (2009). Application of HPLC-NMR for the rapid chemical profiling of a southern Australian sponge, *Dactylospongia* sp.. J. Sep. Sci..

[30] Brkljača R., Gӧker E., Urban S. (2015). Dereplication and chemotaxonomical studies of marine algae of the Ochrophyta and Rhodophyta phyla. Mar. Drugs.

[31] Brkljača R., Dahse H.-M., Voigt K., Urban S. (2019). Antimicrobial evaluation of the constituents isolated from *Macropidia fuliginosa* (Hook.) Druce. Nat. Prod. Commun..

[32] Lever J., Brkljača R., Kraft G., Urban S. (2020). Natural products of marine macroalgae from south eastern Australia, with emphasis on the Port Phillip Bay and Heads regions of Victoria. Mar. Drugs.

[33] Buckle P.J., Baldo B.A., Taylor K.M. (1980). The anti-inflammatory activity of marine natural products—6-n-tridecylsalicylic acid, flexibilide and dendalone 3-hydroxybutyrate. Agents Actions.

[34] Narkowicz C.K., Blackman A.J. (2006). Further acetogenins from Tasmanian collections of *Caulocystis cephalornithos* demonstrating chemical variability. Biochem. Syst. Ecol..

[35] Cho H.-I., Park J.-H., Choi H.-S., Kwak J.H., Lee D.-U., Lee S.K., Lee S.-M. (2014). Protective mechanisms of acacetin against D-galactosamine and lipopolysaccharide-induced fulminant hepatic failure in mice. J. Nat. Prod..

[36] Brkljača R., Urban S. (2015). HPLC-NMR and HPLC-MS profiling and bioassay-guided identification of secondary metabolites from the Australian plant *Haemodorum spicatum*. J. Nat. Prod..

[37] Brkljača R., White J.M., Urban S. (2015). Phytochemical investigation of the constituents derived from the Australian plant *Macropidia fuliginosa*. J. Nat. Prod..

[38] Preston S., Jabbar A., Nowell C., Joachim A., Ruttkowski B., Baell J., Cardno T., Korhonen P.K., Piedrafita D., Ansell B.R.E. (2015). Low cost whole-organism screening of compounds for anthelmintic activity. Int. J. Parasitol..

[39] Tritten L., Silbereisen A., Keiser J. (2011). In vitro and in vivo efficacy of Monepantel (AAD 1566) against laboratory models of human intestinal nematode infections. PLoS Negl. Trop. Dis..

[40] Veglia F. (1916). The anatomy and life history of *Haemonchus contortus* (Rud.). J. Comp. Pathol. Ther..

[41] Weise R.W. (1977). A light and electron microscopic study of the dorsal buccal lancet of *Haemonchus contortus*. J. Parasitol..

[42] Sommerville R.I. (1966). The development of *Haemonchus contortus* to the fourth stage in vitro. J. Parasitol..

[43] Jiao Y., Preston S., Garcia-Bustos J.F., Baell J.B., Ventura S., Le T., McNamara N., Nguyen N., Botteon A., Skinner C. (2018). Tetrahydroquinoxalines induce a lethal evisceration phenotype in *Haemonchus contortus* in vitro. Int. J. Parasitol. Drugs Drug Resist..

[44] Martínez-Ortiz-de-Montellano C., Torres-Acosta J.F.d.J., Fourquaux I., Sandoval-Castro C.A., Hoste H. (2019). Ultrastructural study of adult *Haemonchus contortus* exposed to polyphenol-rich materials under in vivo conditions in goats. Parasite.

[45] Martínez-Ortíz-de-Montellano C., Arroyo-López C., Fourquaux I., Torres-Acosta J.F.J., Sandoval-Castro C.A., Hoste H. (2013). Scanning electron microscopy of *Haemonchus contortus* exposed to tannin-rich plants under in vivo and in vitro conditions. Exp. Parasitol..

[46] Barone C.D., Zajac A.M., Manzi-Smith L.A., Howell A.B., Reed J.D., Krueger C.G., Petersson K.H. (2018). Anthelmintic efficacy of cranberry vine extracts on ovine *Haemonchus contortus*. Vet. Parasitol..

[47] Li X., Deng F., Shan X., Pan J., Yu P., Mao Z. (2012). Effects of the molluscicidal agent GA-C13:0, a natural occurring ginkgolic acid, on snail mitochondria. Pestic. Biochem. Phys..

[48] Jex A.R., Gasser R.B., Schwarz E.M. (2018). Transcriptomic resources for parasitic nematodes of veterinary importance. Trends Parasitol..

[49] Kumarasingha R., Young N.D., Yeo T.-C., Lim D.S.L., Tu C.-L., Palombo E.A., Shaw J.M., Gasser R.B., Boag P.R. (2019). Transcriptional alterations in *Caenorhabditis elegans* following exposure to an anthelmintic fraction of the plant *Picria fel-terrae* Lour. Parasit. Vectors.

[50] Wang T., Ma G., Ang C.-S., Korhonen P.K., Xu R., Nie S., Koehler A.V., Simpson R.J., Greening D.W., Reid G.E. (2019). Somatic proteome of *Haemonchus contortus*. Int. J. Parasitol..

[51] Moser K., Kriwet K., Naik A., Kalia Y.N., Guy R.H. (2001). Passive skin penetration enhancement and its quantification in vitro. Eur. J. Pharm. Biopharm..

[52] Kazlauskas R., Mulder J., Murphy P., Wells R. (1980). New metabolites from the brown alga *Caulocystis cephalornithos*. Aust. J. Chem..

[53] Kelm J.M., Lal-Nag M., Sittampalam G.S., Ferrer M. (2018). Translational in vitro research: Integrating 3D drug discovery and development processes into the drug development pipeline. Drug Discov. Today.

[54] Kamalian L., Chadwick A.E., Bayliss M., French N.S., Monshouwer M., Snoeys J., Park B.K. (2015). The utility of HepG2 cells to identify direct mitochondrial dysfunction in the absence of cell death. Toxicol. In Vitro.

[55] Liu Z.H., Zeng S. (2009). Cytotoxicity of ginkgolic acid in HepG2 cells and primary rat hepatocytes. Toxicol. Lett..

[56] Zhou C., Li X., Du W., Feng Y., Kong X., Li Y., Xiao L., Zhang P. (2010). Antitumor effects of ginkgolic Acid in human cancer cell occur via cell cycle arrest and decrease the Bcl-2/Bax ratio to induce apoptosis. Chemotherapy.

[57] Wu L., Jiang X.G., Shen Y.J., Lu Z.X., Tu G.H., Fu X.L., Chen S.X., Cao J.P. (2011). Efficacy of ginkgolic acids against *Cryptosporidium andersoni* in cell culture. Parasitol. Res..

[58] Ugwu C.E., Jiang Y.Y., Wu L., Xu Y.X., Yin J.H., Duan L.P., Chen S.X., Liu H., Pan W., Quan H. (2019). In vitro Screening of ginkgolic acids for antiparasitic activity against *Cryptosporidium andersoni*. Biomed. Environ. Sci..

[59] Lü J.-M., Yan S., Jamaluddin S., Weakley S.M., Liang Z., Siwak E.B., Yao Q., Chen C. (2012). Ginkgolic acid inhibits HIV protease activity and HIV infection in vitro. Medical Sci. Monit. Int. Medical J. Exp. Clin. Res..

[60] Zhang Z.-B., Ruan C.-C., Chen D.-R., Zhang K., Yan C., Gao P.-J. (2016). Activating transcription factor 3 SUMOylation is involved in angiotensin II-induced endothelial cell inflammation and dysfunction. J. Mol. Cell Cardiol..

[61] Qiu F., Dong C., Yanxin L., Shao X., Huang D., Han Y., Wang B., Liu Y., Huo R., Paulo P. (2018). Pharmacological inhibition of SUMO-1 with ginkgolic acid alleviates cardiac fibrosis induced by myocardial infarction in mice. Toxicol. Appl. Pharm..

[62] Baek S., Lee J., Kim C., Ko J.-H., Ryu S.-H., Lee S.-G., Yang W., Um J.-Y., Chinnathambi A., Alharbi S. (2017). Ginkgolic acid C 17:1, derived from *Ginkgo biloba* leaves, suppresses constitutive and inducible STAT3 activation through induction of PTEN and SHP-1 tyrosine phosphatase. Molecules.

[63] Kleijnen J., Knipschild P. (1992). *Ginkgo biloba*. Lancet.

[64] Chassagne F., Huang X., Lyles J.T., Quave C.L. (2019). Validation of a 16th century traditional Chinese medicine use of *Ginkgo biloba* as a topical antimicrobial. Front. Microbiol..

[65] Ude C., Schubert-Zsilavecz M., Wurglics M. (2013). *Ginkgo biloba* extracts: A review of the pharmacokinetics of the active ingredients. Clin. Pharmacokinet..

[66] Liu H., Ye M., Guo H. (2020). An updated review of randomized clinical trials testing the improvement of cognitive function of *Ginkgo biloba* extract in healthy people and Alzheimer’s patients. Front. Pharmacol..

[67] Schwarz E.M., Korhonen P.K., Campbell B.E., Young N.D., Jex A.R., Jabbar A., Hall R.S., Mondal A., Howe A.C., Pell J. (2013). The genome and developmental transcriptome of the strongylid nematode *Haemonchus contortus*. Genome Biol..

[68] Kaminsky R., Ducray P., Jung M., Clover R., Rufener L., Bouvier J., Weber S.S., Wenger A., Wieland-Berghausen S., Goebel T. (2008). A new class of anthelmintics effective against drug-resistant nematodes. Nature.

[69] Rufener L., Bedoni N., Baur R., Rey S., Glauser D.A., Bouvier J., Beech R., Sigel E., Puoti A. (2013). *acr-23* encodes a monepantel-sensitive channel in *Caenorhabditis elegans*. PLoS Pathog..

[70] Prichard R.K., Geary T.G. (2019). Perspectives on the utility of moxidectin for the control of parasitic nematodes in the face of developing anthelmintic resistance. Int. J. Parasitol. Drugs Drug Resist..

