# Supplementary material for: Natural Compounds from the Marine Brown Alga Caulocystis cephalornithos with Potent In Vitro-Activity against the Parasitic Nematode Haemonchus contortus
Source: Pathogens. 2020 Jul 9;9(7):550. doi: 10.3390/pathogens9070550 (PMC7400099; doi:10.3390/pathogens9070550)
Supplement: Supplementary file 1 [file pathogens-09-00550-s001.pdf]

Supplementary File 1

Natural Compounds from the Marine Brown Alga *Caulocystis cephalornithos* with Potent *In Vitro*-Activity against the Parasitic Nematode *Haemonchus contortus*

Aya C. Taki [1], Robert Brkljača [2,3], Tao Wang [1], Anson V. Koehler [1], Guangxu Ma [1], Jill Danne [4], Sarah Ellis [4], Andreas Hofmann [1], Bill C. H. Chang [1], Abdul Jabbar [1], Sylvia Urban [2,*] and Robin B. Gasser [1,*]

[1] Department of Biosciences, Melbourne Veterinary School, Faculty of Veterinary and Agricultural Sciences, The University of Melbourne, Parkville, Victoria 3010 Australia; aya.taki@unimelb.edu.au (A.C.T.); tao.wang1@unimelb.edu.au (T.W.); anson.koehler@unimelb.edu.au (A.V.K.); guangxum@student.unimelb.edu.au (G.M.); a.hofmann@structuralchemistry.org (A.H.); bchang@yourgene.com.tw (B.C.H.C.); jabbara@unimelb.edu.au (A.J.)

[2] School of Science (Applied Chemistry and Environmental Science), RMIT University, Melbourne, Victoria 3001, Australia;

[3] Monash Biomedical Imaging, Monash University, Clayton, Victoria 3168 Australia; robert.brkljaca@monash.edu (R.B.)

[4] Peter MacCallum Cancer Centre, Melbourne, Victoria 3010 Australia; jill.danne@petermac.org (J.D.); sarah.ellis@petermac.org (S.E.)

* Correspondence: robinbg@unimelb.edu.au (R.B.G.); sylvia.urban@rmit.edu.au (S.U.)

Supplementary Methods

<u>The collection, extraction and purification of compounds 1 and 2:</u>

Collection: A sample of the southern Australian marine sponge *Dactylospongia* sp. (voucher code 2004_48) was collected by Mr Richard Goudie at several sites in Gunnamatta Beach, Victoria on the 26/11/2004. The sponge was collected via diving using self-contained underwater breathing apparatus (SCUBA) at a depth of 8 to 15 metres and identified by Ms Lisa Goudie as Demospongiae, Dictyoceratida, Thorectidae, *Dactolospongia* sp.

Extraction: The marine sponge specimen 2004_48 (80 g, wet weight) was extracted using 3:1 methanol (MeOH)/dichloromethane (DCM) to yield 1.5 g of a brown gum. The gum was sequentially solvent partitioned into DCM followed by MeOH respectively.

Semi-preparative reversed phased HPLC: Reversed phased semi-preparative HPLC was carried out on the DCM extract (960 mg) on a Dionex P680 (solvent delivery module) equipped with a Dionex UVD340U PDA detector and a Foxy Jr. automated fraction collector. An isocratic method (100% CH_3CN) was employed using a Phenomenex Luna (2) 100 Å C_{18} 250 x 10 mm (5 μm) column. The automatic fraction collector was programmed to collect based on elution time and a peak threshold. This resulted in the isolation of the compounds **1** (35.8 mg) and **2** (49.8 mg).

<u>The collection, extraction and purification of compounds 3 and 4:</u>

Collection: The sample of the brown alga *Caulocystis cephalornithos* was collected by Dr Sylvia Urban on the 22nd of January 2006 intertidally from the Borough of Queenscliff, Victoria. The alga was assigned

a unique voucher code of 2006-07 and was subsequently identified by Dr Gerald Kraft (The University of Melbourne, Australia).

Extraction: The *C. cephalornithos* specimen (240 g, wet weight) was extracted using 3:1 MeOH/DCM, followed by trituration into DCM and MeOH (soluble extracts).

Column Chromatography: The DCM extract (0.77 g), was subjected to silica flash chromatography using a 20% stepwise elution profile from 100% petroleum spirits followed by DCM, ethyl acetate (EtOAc) and finally to MeOH. Following collection of the 100% MeOH fraction a final elution was performed using 100% MeOH containing 0.1% trifluoracetic acid (TFA) to yield a total of 11 fractions. The fourth fraction was purified further by reversed phase semi-preparative HPLC using an isocratic solvent composition of 90% MeOH/water (0.1% TFA) monitoring at a wavelength of 275 nm to yield 6-tridecylsalicylic acid (**3**) (14.1 mg) and 6-undecylsalicyclic acid (**4**) (5.0 mg).

The collection, extraction and purification of compound 5:
For details about the collection, extraction and compound purification, refer to ref. [36].

The collection, extraction and purification of compounds 6 and 7:
For details about the collection, extraction and compound purification, refer to ref. [37].

The collection, extraction and purification of compound 8:
Collection: The medicinal plant *A. rugosa* was collected in March 2012 by Dr Sylvia Urban and Professor Eddie Pang at RMIT University, Australia. Professor Pang assisted in identifying the species. The plant was collected at the Chinese medicinal plant garden at the RMIT Bundoora west campus and each plant was assigned a unique voucher code of 2012-03.

Extraction: Flowers of *A. rugosa* (65 g, dry weight) were extracted in 3:1 DCM/MeOH in 2 L of solvent over two weeks. The crude extract was then sequentially partitioned into DCM and MeOH fractions, respectively.

Chromatography: The compounds in the DCM fraction (868 mg) were separated by a flash silica column to give a total of fifteen fractions. Fraction ten, which was eluted from the silica column using 40% DCM and 60% EtOAc, was subjected to semi-preparative HPLC and performed on a Varian Pro Star 210 solvent delivery system, using a Phenomenex C18 100 Å 250 x 10 mm (5 μ) column with a Varian Pro Star 335 PDA detector at a flow rate of 3.5 mL/min using 50% CH_3CN/H_2O resulting in the isolation of compound **8** (1.3 mg).

Structure determination and purity verification of isolated natural products: All natural products isolated were elucidated via NMR spectroscopy (1D and 2D) and mass spectrometry and confirmed by comparison to data reported in the literature. ^1H (500 MHz), ^{13}C (125 MHz), and 1D NOE spectra were acquired in $CDCl_3$ and DMSO-d_6 on a 500 MHz Agilent DD2 NMR spectrometer with referencing to solvent signals (δ_H 7.26; δ_C 77.0 and δ_H 2.50; δ_C 39.5 ppm, respectively). Two-dimensional NMR

experiments employed gradient correlation spectroscopy (gCOSY), heteronuclear single-quantum correlation spectroscopy with adiabatic pulses (HSQCAD), and gradient heteronuclear multiple-bond spectroscopy with adiabatic pulses (gHMBCAD). ESI mass spectra were obtained using a Micromass Platform II equipped with an LC-10AD Shimadzu solvent delivery module (50% CH_3CN/H_2O at a flow rate of 0.2 mL/min) in both the positive and negative ionization modes using cone voltages between 20 and 30 V. Compound purity was assessed by reanalysis via analytical HPLC which was performed on a Dionex P680 solvent delivery system equipped with a PDA100 UV detector (operated using Chromeleon software). Analytical HPLC analyses were carried out using either a gradient method (0−2 min 10% CH_3CN/H_2O; 14−24 min 75% CH_3CN/H_2O; 26−30 min 100% CH_3CN; and 32−40 min 10% CH3CN/H_2O or 0−3.5 min 30% CH_3CN/H_2O; 8.5−40 min 50% CH_3CN/H_2O) or an isocratic method (either 70, 50, 45, 40, 30, or 25% CH_3CN/H_2O) on an Alltech Alltima HP C18 (250 × 4.6) 5 μm column at a flow rate of 1.0 mL/min.
